# Recent Advances in Heterogeneous Photocatalytic Decolorization of Synthetic Dyes

**DOI:** 10.1155/2014/692307

**Published:** 2014-06-25

**Authors:** Nurhidayatullaili Muhd Julkapli, Samira Bagheri, Sharifah Bee Abd Hamid

**Affiliations:** Nanotechnology & Catalysis Research Centre (NANOCAT), IPS Building, University Malaya, 50603 Kuala Lumpur, Malaysia

## Abstract

During the process and operation of the dyes, the wastes produced were commonly found to contain organic and inorganic impurities leading to risks in the ecosystem and biodiversity with the resultant impact on the environment. Improper effluent disposal in aqueous ecosystems leads to reduction of sunlight penetration which in turn diminishes photosynthetic activity, resulting in acute toxic effects on the aquatic flora/fauna and dissolved oxygen concentration. Recently, photodegradation of various synthetic dyes has been studied in terms of their absorbance and the reduction of oxygen content by changes in the concentration of the dye. The advantages that make photocatalytic techniques superior to traditional methods are the ability to remove contaminates in the range of ppb, no generation of polycyclic compounds, higher speed, and lower cost. Semiconductor metal oxides, typically TiO_2_, ZnO, SnO, NiO, Cu_2_O, Fe_3_O_4_, and also CdS have been utilized as photocatalyst for their nontoxic nature, high photosensitivity, wide band gap and high stability. Various process parameters like photocatalyst dose, pH and initial dye concentrations have been varied and highlighted. Research focused on surface modification of semiconductors and mixed oxide semiconductors by doping them with noble metals (Pt, Pd, Au, and Ag) and organic matter (C, N, Cl, and F) showed enhanced dye degradation compared to corresponding native semiconductors. This paper reviews recent advances in heterogeneous photocatalytic decolorization for the removal of synthetic dyes from water and wastewater. Thus, the main core highlighted in this paper is the critical selection of semiconductors for photocatalysis based on the chemical, physical, and selective nature of the poisoning dyes.

## 1. Introduction

### 1.1. Photocatalytic Decolorization in Water and Wastewater Treatment

**Figure 1:**
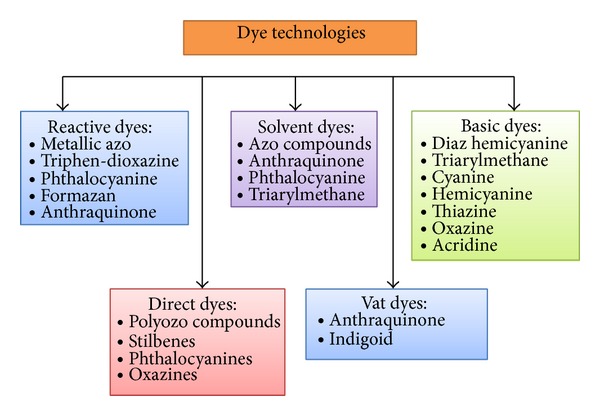
Synthetic dyes and its derivatives.

Generally, dyes are complex unsaturated aromatic compounds with accomplishing characteristics like color, intensity, solubility, fastness, and substantiveness [1, 2]. It could be compounds with different coloring particles, each varying in type from each other in terms of chemical composition, and are used for coloring textiles in different colors and shades that are completely soluble in aqueous media [2, 3]. Dyes derived from inorganic or organic compounds are called synthetic dyes and they are categorized based on their basic chemistry (Table 1; Figure 1). There are various ways used for the assortment of dyes. It should be noted that each category of dyes has an exclusive chemistry, source of materials, nature of its respective chromophores, nuclear structure, industrial classification, and specific way of bonding. Although some dyes can chemically react with the substrates forming robust bonds in the process, others can be sustained by physical forces. The most common synthetic dyes in use today are dispersible types for polyester dyeing and reactive and direct types for cotton dyeing.

Synthetic dyes are also utilized in high technology applications, like in the electronics, medical, and specifically the nonimpact printing industries. For instance, they are utilized in electrophotography (laser printing and photocopying) in both the organic photoconductor and the toner, in direct and thermal transfer printing, and also in ink-jet printing. With increasing synthetic dye usage, dye removal becomes an important but challenging area of research for wastewater treatment since most of dyes and their degradation products may be carcinogenic and toxic to mammals [4, 5].

Heterogenous photocatalysis using semiconductors for water and wastewater treatment continues to attract much interest [4, 5, 37, 38]. The lower cost of catalysts and the utilization of environmental protection and renewable energy form this technology to be adequately attractive compared to other techniques [37]. Because the process relies on the photoactivation of semiconductors, the efficiency of the catalyst is qualified by the capacity to generate electron-hole pairs in addition to radical production [39, 40]. Hence, the selection of proportionate semiconductors is the key to reactivity control [38].

### 1.2. Poisoning Dyes

Only 45 to 47% of dyes have been reported as organic dyes with biodegradable and solubility characteristics. The remaining 55 to 53% of dyes are toxic and their persistence in wastewater has recently become an issue of interest [8, 41, 42]. Synthetic or poisoning dyes engaged more often on industrial scale are acid dyes, water soluble anionic, basic dyes-water soluble cationic, substantive dyes-alkaline, vat dyes-water soluble alkali metal salt, azoic dyes, sulfur dyes, and chrome dyes. Generally, there are two important components in the dye molecules: chromophore component that is responsible for producing the color and the auxochrome component which increases the affinity of the dye towards cellulose fibers [14, 43].

The mentioned dyes are released in aqueous streams as effluents of several industries, including textiles, paper, leather, plastic, automobile, furniture, finishing sector, and others, which consequently create intense environmental pollution problems via the release of potential carcinogenic and toxic substances into the aqueous phase [22, 23]. The discharge of an enormous volume of wastewater containing dyes is an inevitable consequence, because the textile industry consumes large quantities of water and all dyes cannot be completely combined with fibers during the dyeing process. More than 79105 metric tonnes of dye stuffs are produced worldwide annually, with 10 to 50% of this amount being released into wastewater [18, 44]. These high concentrations of dyes in effluents interfere with the penetration of visible light into the water, resulting in a hindrance to photosynthesis and a decrease in gas solubility, since less than 1 mgL^−1^ of dye is highly visible. Furthermore, synthetic dyes, which include an aromatic ring in their basic structure, are regarded as toxic, carcinogenic, and xenobiotic compounds [43–46]. Also, this type of dyes may convey toxicity to aquatic life and may be mutagenic and carcinogenic and can cause intense damage to human beings, including the reproductive system and dysfunction of the kidneys, brain, liver, and central nervous system [34].

Therefore, decolorization and detoxification of dye-containing wastewater need to be conducted before discharging wastewater into natural water bodies [26, 27, 29]. Certain physical, chemical, and biological treatments are currently being used for dye wastewater treatment. Although physical and chemical methods usually show high dye-removal efficiencies, high operating costs are the main drawback due to the large-scale application of these methods [32, 47, 48]. Furthermore, due to the high chemical stability of synthetic dyes, conventional biological treatment using bacteria cannot remove the dyes efficiently [43, 49, 50].

## 2. Photocatalytic Decolorization of Synthetic Dyes

The complete degradation of the dyes is not possible by conventional methods such as precipitation, adsorption, flocculation, flotation, oxidation, reduction, electrochemical, aerobic, anaerobic, and biological treatment methods. These methods have inherent limitations in technologies such as less efficiency and production of secondary sludge, the disposal of which is a costly affair [43–53]. Merely, transferring hazardous materials from one medium to another is not a long-term solution to the issue of toxic waste loading on the environment [30]. Many technologies have been applied to remedy dyes from wastewater, like coagulation/flocculation, biological treatment, electrochemical, membrane filtration, ion exchange, adsorption, and chemical oxidation [54, 55]. Chemical coagulations for dye removal require loading of chemical coagulation and optimal operating conditions like pH and coagulation dosage should be rigidly reminded for achieving maximum dye removal [56]. The coagulation-flocculation process can be utilized as a pre- or post- or even as a main treatment. This process is cost effective and easy as it consumes less energy than the conventional coagulation treatment [57]. However, utilizing inorganic salts like aluminum chloride and aluminum sulfate as the coagulation agent has now become controversial because of their possibility of contributing to Alzheimer's disease [56–58]. Polyacrylamide-based materials are also often utilized in the coagulation process, but the possible release of monomers is now considered damaging due to their entering into the food chain and causing potential health impacts (e.g., carcinogenic effects).

Adsorption removal method is a simple and effective method/design since it is easy to use and can be implemented for dye treatment even in small plants; however, it usually produces huge amounts of sludge, especially in the wastewater with high dye concentrations [59]. Adsorption of dyes on many adsorbents (e.g., SiO_2_, Al_2_O_3_, CaO, MgO, Fe_2_O_3_, Na_2_O, K_2_O, bentonite, and montmorillonite) has been broadly studied, but the activated carbon has been proven to be the most effective catalyst due to its high specific surface area, ultra high adsorption capacity, and low selectivity for both nonionic and ionic dyes. However, it has some limitations, including the need for regeneration after exhausting, high cost of the activated carbon, and the lack of adsorption efficiency after regeneration [59, 60]. Taking all these facts into consideration, much of the present work involves the degradation and mineralization of synthetic dyestuff in industry by heterogenous photocatalyst.

The heterogenous photocatalyst relates to the water decontamination processes that are concerned with the oxidation of biorecalcitrant organic compounds [4, 61, 62]. This impressive method relies on the formation of highly reactive chemical species that degrade a number of recalcitrant molecules into biodegradable compounds and is known as the advanced oxidation process (AOP).

The Environmental Protection Agency (EPA) has approved the inclusion of AOP as the best available technology to meet the standard with specifications that provide safe and sufficient pollution control of industrial processes and remediation of contaminated sites [42, 63].

**Figure 2:**
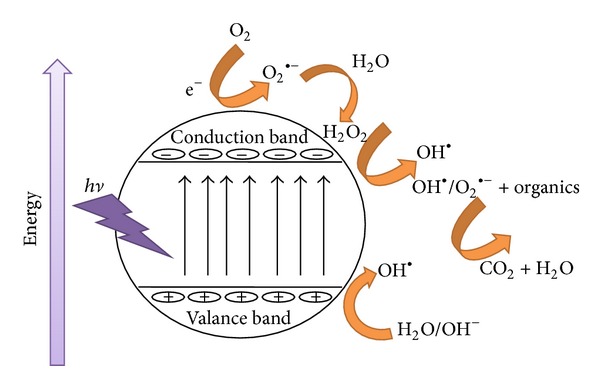
General view on photocatalytic mechanism and degradation process.

Advanced oxidation processes are based on the production of hydroxyl radicals which oxidize a wide range of organic pollutants including dyes quickly and nonselectively. AOPs include homogenous and heterogeneous photocatalytic oxidation systems. The homogenous photocatalytic oxidation system employs various oxidants such as H_2_O, O_3_, Fenton reagent, NaOCl, and many others either alone or in combination with light [64] (Figure 2). Recently, heterogeneous photocatalysis has emerged as an important degradation technology leading to the total mineralization of organic pollutants, especially synthetic dyes [5, 37, 38, 65, 66].

### 2.1. Photocatalytic Decolorization of Acid Dyes-Water Soluble Anionics

Acid dyes are chemically a sodium (less often ammonium) salt of a carboxylic or phenol organic acid, or sulfuric acid, with ionic substitution to be soluble in water and contains affinity for amphoteric fibers, while lacking direct dye affinity for cellulose fibers (via hydrogen bonding, Van de Waals, and ionic bonding) [67, 68]. Acid dyes consist of several compounds from the most varied categories of dyes, which represent characteristic differences in structure (e.g., nitro dyes, triphenylmethane, and anthraquinone) [69]. Acid dyes are commonly divided into several classes which depend on level dyeing properties, fastness requirements, and economy, which are indicated by the strength of the anionic characteristic of dyes to the cationic sites of the cellulose fibers [68]. Most of acid dyes are generated from chemical intermediates, where anthraquinone-like structures and triphenylmethane predominate as the final state, which give blue, yellow, and green color [68–70].

Acid dyes, just as any of the synthetic dyes, have the capability of persuading sensitization in humans because of their complex molecular structure and the way in which they are metabolized in the body. Moreover, their water solubility is harmful to human beings since they are sulphonic acids [71]. The sulphonate groups are spread evenly along the molecule on the opposite side to the hydrogen bonding –OH groups, to minimize any repulsive effect [69]. This in consequence determines the main problem with anionic dyes, which is the lack of fastness during the washing and removing process.

Thus, many research works have paid increasing attention to the degradation of acid dyes in the water stream in recent years. Several techniques, including the use of activated carbon, membrane filteration, adsorption, and coagulation have been known to unravel the problems caused by the presence of acid dyes (Table 2).

**Figure 3:**
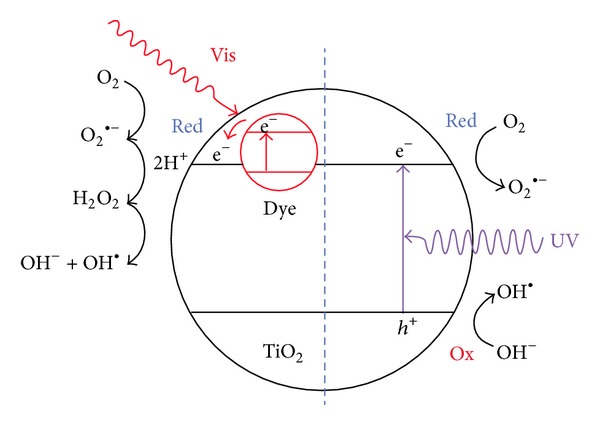
The photocatalytic decolorization of TiO_2_ towards Acid Red 44 as a model of acid dyes [83].

However, due to the recalcitrant nature of acid dyes and the high salinity of wastewater containing acid dyes, these conventional treatment processes are feckless. Adsorption and coagulation methods have also been applied to treat acid dyes in wastewater, which always result in secondary pollutants [66]. Furthermore, it is noted that acid dyes have –SO^3−^, –COOH, –OH, and hydrophilic groups and excellent solubility in the water stream [74, 75]. Their molecules spread linearly in solution and have a notable tendency to aggregate by hydrogenous bonding, and consequently form colloids in solution and also tend to be adsorbed and flocculated [81]. To overcome such limitations, photocatalytic decolarization of acid dyes water soluble is essential. This process done through the formation of electron-hole pairs with proper photon energy. It has been assumed that once the energy is larger than the band gap, the electron-hole pairs are separated between the semiconductor's valence and conduction bands [61, 82]. The acid dyes as adsorbed species on suitable sites on the surface of semiconductors undergo photooxidation, reduction, and synthesis under either ultraviolet, sunlight, or even ultrasonic lights. In addition, the aromatic linkages are susceptible to reduction under light irradiation [83] (Figure 3).

This encourages a promising technology based on the advanced oxidation process that has been studied extensively through a broad range of acid dyes that can be nonselectively oxidized quickly [43, 84, 85]. Photocatalysis of acid dyes entails the formation of adequate concentrations of highly reactive transitory species like hydrogen peroxide, hydroxyl radicals, and superoxides to react with acid dyes and degrade them in the presence of a semiconductor and visible light or ultraviolet (UV) light [86]. Usually the semiconductors with band gap energy of 3.2 eV are used as photocatalysts with the assumption that as a proton at equal or higher energy (*λ* < 400 nm) illuminates the semiconductor, the photon energy creates an electron to jump from the valence band to the conduction bands, generating electrons and positively charged holes [51, 87]. These electron-hole pairs persuade a series of reactions, which oxidize the dye acids.

Among the various semiconductor oxides, TiO_2_ and ZnO have been intensively investigated since the discovery of their ability to photocatalyse acid dyes [37]. Briefly, once the aqueous semiconductor (TiO_2_ and/or ZnO) suspensions are irradiated in light energy greater than the band gap energy of the semiconductors, conduction band electrons and valence band holes are generated [51, 88, 89]. As the charge separation is maintained, the electrons and holes may migrate to the semiconductor surface where it takes part in the redox reaction with acid dyes [90–92]. The photogenerated electrons react with the adsorbed acid dye molecules (O_2_) on the semiconductor site and diminish it to superoxide radical anion (O_2_^•^) while the photogenerated holes oxidize the H_2_O or OH^−^ ions adsorbed at the semiconductor surface to OH^•^ radicals [43, 93–95]. These generated radicals with other highly oxidant species act as strong oxidizing agents which could easily attack the adsorbed acid dye molecules or those located close to the surface of the semiconductor, thus resulting in complete degradation of acid dyes into its smaller biodegradable fragments [89, 96].

Despite the many benefits of using TiO_2_ and ZnO as a photocatalyst to degrade the dye acids, if the aim is to expand a solar-powered treatment technology, there are few disadvantages of the technology that barricade commercialization. Even if both semiconductors offer high absorption and surface areas, they can be adjusted by preparation parameters [84, 97, 98]. Although many acid dyes can be effectively photodecomposed using TiO_2_ and/or ZnO as the photocatalyst, the kinetics and mechanism of photocatalytic decolorization with respect to both semiconductors as photocatalysts are comparatively unclear. It has been recorded that both semiconductors can contribute to the decomposition reaction in different ways without decreasing their activity over time [99]. Several kinetic models for catalyzed oxidation utilizing heterogenous catalyst supported by both organic and inorganic carriers have been published in the literature [51, 83, 100]. However, only a few kinetic models of catalyzed photocatalytic decolorization of acid dyes were published. The Mars-Van Krevelen mechanism stated that the surface of the semiconductor catalyst acted as redox mediator, which transferred electrons to oxygen to form oxygen anions as radicals, O_2_^−•^. The O_2_^−•^ anion radical oxidized the adsorbed acid dye compounds to form various products, while the reduced form of O_2_^−^ could be regenerated by gaseous oxygen [61, 101]. The stationary-state adsorption mechanism was based on the steady-state assumption and also the oxidation reduction of the adsorbed phase [102]. The Ely-Rideal mechanism envisaged that a heterogeneous reaction took place among strongly chemisorbed acid dye atoms and physically adsorbed molecules which become attached to the surface by faint Van der Waals forces [84]. The Langmuir-Hinshelwood mechanism is based on the reaction that occurred between both acid dyes and semiconductors [95, 103].

### 2.2. Photocatalytic Decolorization of Basic Dyes-Water Soluble Cationics

**Figure 4:**
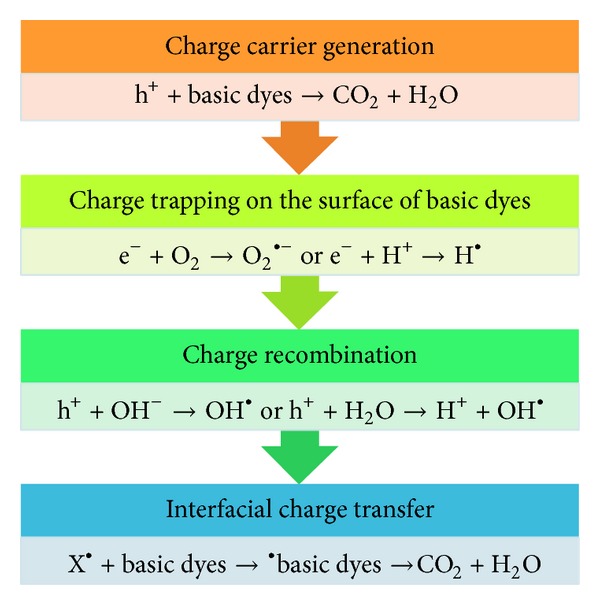
The steps in the photocatalytic process of basic dyes using TiO_2_ or ZnO.

Water soluble basic dyes are commonly considered as the most difficult to eliminate or degrade from the dyeing effluent, because of their high stability and resistance ability in the water stream [104–106]. Basic dyes possess cationic functional groups such as –NH^3+^ or =NR^2+^ [105]. Both of these protein functional groups in basic conditions generate a negative charge as the –COOH groups are deprotonated to give –COO^−^ [107]. Basic dyes perform weakly on natural fibers but work very well in acrylics [105]. Basic dyes will form a covalent bond with the proper polyacrylic functionality, and once attached, these basic dyes are very difficult to remove [106]. Cationic triphenylmethane dyes are one of the most extensive basic dyes utilized as colorants and antimicrobial agents in different industries. Previous articles demonstrate that it may further serve as targetable sensitizers in photodestruction of specific cellular components or cells [107, 108]. Methyl green (MG) is a basic triphenylmethane and dicationic dye frequently utilized for staining of solutions in biology and medicine. It is also utilized as a photochromophore to sensitize gelatinous films [109]. The increasing interest in the development of modern and new methodologies for the degradation of toxic basic dyes has led to the deduction that the most effective way for oxidation of the basic dyes is with a powerful oxidizing agent, specifically when a free radical like ^•^OH is generated [110–112] (Figure 4).

Lately, advanced oxidation processes have been broadly investigated and have become alternative methods for decolorizing and reducing recalcitrant wastewaters generated by basic dyes. Likewise, the use of cadmium oxide (CdO) nanostructure as one of the promising semiconductors for this operation demonstrates positive results [113–115]. CdO is an n-type semiconductor with a direct band gap of 2.2 to 2.5 eV and an indirect band gap of 1.36 to 1.98 eV [114]. Since CdO has a band gap tailored to the visible region of solar light with a similar photocatalytic mechanism to semiconductor oxides, it can be an important option as photocatalyst materials especially in the decolorization process of basic dyes [45, 113]. Indeed, the evaluation of photocatalytic activity of CdO towards basic dyes is considered as cauliflower-like [116]. The nanostructure of CdO for removing the basic dyes from aqueous solution has been reported and it is believed that the crystal orientation, morphology, crystallinity, particle size, architecture types, and oxygen defects play an important role in changing the band gap. Actually, diversity in the band gap energy is highlighted to lattice defects because of the Burstein-Moss effect. Besides, the catalytic, optical, and electrical properties originate from the difference of band gaps in different structures [115]. Thus, it is critical to probe an investigation on the generation of new CdO structures for better photodegradation of basic dyes. Different structures of CdO on a nanoscale have been reported, such as nanowires, nanoparticles, nanoneedles, thin film, nanocrystal, and others [117]. CdO micro- and nanoarchitectures with three-dimensional structures such as rods, tubes, and cauliflower-like structures have a larger specific surface area and enhanced oxygen vacancy, which in turn increases the degree of oxidation process on basic dyes [118]. Cauliflower-like architectures have attracted great interest due to its special and novel morphology with high specific surface area that can facilitate the diffusion and mass transportation of the basic dye molecules in photodegradation applications [116]. This particular structure can be easily synthesized using mechanochemical methods, a cheap process, followed by thermal treatment conforming to the detailed process presented in former studies.

Most studies related to photodegradation techniques have been done using TiO_2_ and/or ZnO as the model photocatalyst because of their nontoxicity, cheapness, chemical stability, and high photocatalytic activity [37, 119–121]. The photocatalytic decolorization of basic dyes with TiO_2_ and/or ZnO as the charge carrier or generation is summarized in Figure 2. The OH^•^ or the directly produced charge is a strong oxidizing agent which attacks basic dyes present at or near the surface of the semiconductor [122]. It ultimately causes the complete degeneration of the basic dyes into harmless compounds. In general, two different types of TiO_2_ phase are normally used in photocatalytic decolorization of basic dyes: anatase (3.2 eV) and rutile (3.0 eV). The adsorptive affinity of anatase for the basic dyes is higher than that of rutile, and thus anatase is generally regarded as the more photocatalytic active phase of TiO_2_, presumably due to the combined effect of lower rates of recombination and higher surface sites [123, 124].

The dye derivative reactive brilliant blue (KN-R) has been broadly utilized as a model of basic dyes in the photocatalysis process. The effects of key operational factors like reaction pH, catalyst loading, H_2_O_2_ dosage, and the initial basic dye concentration on the decolorization were extensively studied to optimize the process for maximum degradation of basic dyes [125]. It can be concluded that the photocatalytic decolorization process performed a fast oxidation without the formation of polycyclic products and intermediate products at a suitable wavelength of light [51, 126]. The reactions frequently take place on the surface of the semiconductors. Hence, the need for a semiconductor supported by a good adsorbent is much felt because of the power to concentrate pollutants near semiconductor particles and the capacity for adsorption of generated intermediates and the capability of reusing adsorbents [127]. In addition, to ensure full use of the solar energy source, it is of great interest to develop photocatalysis of basic dyes for expansion of the adsorption to the visible light range. For both TiO_2_ and/or ZnO, a great deal of effort has been focused to extend their photoadsorption to the visible light range, for example, by doping with anions of C, S, and N or transition metal cations [128, 129]. Besides TiO_2_ and/or ZnO, a great deal of attention has also been focused in the search for semiconductor oxides of Bi_2_WO_6_, BiM_ox_O_6_, Bi_2_M_ox_W_1−*x*_, and Bi_4_Ti_3_O_12_ which have been recently revealed to exhibit photocatalytic activity and decolorization of basic dyes in the visible light range owing to their lower band gap than that of TiO_2_ and/or ZnO [130–140].

### 2.3. Photocatalytic Decolorization of Disperse Dyes—Alkaline

Disperse dyes have low solubility in water. However, they can interact with the polyester chains by forming dispersed particles. Their main application is the dyeing of polyester, and they find less use in dyeing cellulose acetates and polyamides [141–145]. The general structure of disperse dyes is planar, small, and nonionic, with attached polar functional groups such as –NO_2_ and –CN. In addition, this type of dyes is a mitotic toxication agent and should be considered as a biohazard component [142]. Thus, discharge of disperse dyes have become a subject of concern in the universe due to its harmful and toxic effects to living organisms and the environment [143]. As far as the wastewater treatment technologies are concerned, different techniques have been utilized for the reduction and degradation of dispersed dyes such as chemical precipitation, H_2_O_2_ adsorption, oxidation by chlorine, electrochemical treatment, ozone electrolysis, adsorption, ion pair extraction, flocculation, coagulation, membrane filtration, and specially the photocatalytic process [145–147].

**Figure 5:**
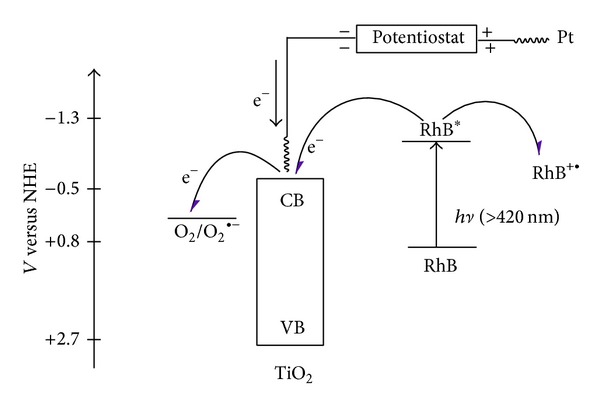
Proposed mechanism of the photoelectrocatalytic degradation of Rhodamine B with TiO_2_ as the electrode [155].

The dispersed dyes (alkaline compounds) can be most effectively decomposed by photocatalytic methods [148–150]. Recently, owing to their unique and special electrical and optical properties, semiconductor materials have gained global acceptance for alkaline dispersed dye treatments [151]. It has been demonstrated that the photooxidation of CN to OCN occured during the photodegradation of alkaline dyes in the presence of powerful oxidation agents [152–154]. Considering that disperse alkaline dyes cannot be treated by conventional biological processes, intensive investigations on the latest treatment techniques of these wastewaters have been conducted to develop effective methods for the remediation and treatment of a wide variety of alkaline-dye pollutants owing to their capability to produce a complete degradation process. The photocatalytic degradation reaction is usually conducted for compounds dissolved in water-like alkaline dyes, at mild temperature and pressure conditions, utilizing ultraviolet-illuminated semiconductor powders without the requirements of expensive oxidants [86, 155–158] (Figure 5).

A semiconductor is generally characterized by the band gap energy between its electronically populated valence band and its broadly vacant conduction band [33].

Copper oxide (CuO) is a one of the most promising semiconductors used in advanced oxidation processes for degradation of alkaline dyes [159–161]. With an energy band gap of 1.21 to 1.5 eV it has the ability to perform under irradiation in sunlight. Reactions involving Cu^+^/Cu^2+^ lead to the oxidative transformation of alkaline dyes. The unique electronic structure of Cu allows for the interaction with the spin restricted O_2_ enabling Cu to participate in the redox reaction with alkaline dyes [162]. Many researchers have anticipated the reaction of CuO on different adsorbents like activated alumina, zeolite, or activated carbon in wastewater treatments. It was found that in order to achieve an efficient, stable, and economical catalyst, CuO semiconductors must be fixed on an ideal and an inert support [163–165]. Among all CuO supported systems for alkaline dye photodecolorization, zeolite was found to be the most ideal with several distinct advantages, including super adsorption capability, unique uniform pores, and special ion-exchange capability [166].

### 2.4. Photocatalytic Decolorization of Vat Dyes-Water Soluble Alkali Metal Salts

Almost 22% of the total volume of industrial wastewater produced comes from the textile industry, with 7 × 10^5^ tonne of materials classified as vat dyes or water soluble alkali metal salts [167, 168]. This type of dyes produces undesirable effluents and is discharged into the environment without further treatment. Once the vat dyes enter natural water bodies, it can cause intense problems if not treated, since the dyes are toxic, mutagenic, and carcinogenic to human life as well as can inhibit photosynthesis of aquatic life even in quantities as low as 1 ppm [169]. To solve this problem, several semiconductors for oxidative photodecolorization have been tested, including WO_3_, TiO_2_, MnO, CuS, ZnO, Fe_2_O_3_, ZrO_2_, CuO, CdS, ZnS, In_2_O_3_, SnO_2_, and Nb_2_O_5_ [170–176].

The selection of the type of semiconductor is based on its ability to convert the vat dyes into nontoxic products [44, 90, 177, 178]. Additionally, the use of mesoporous materials, like zeolite as a support for these series of semiconductors, has recently become the focus of intensive research on vat dye photodecolorization, due to the fact that the semiconductor support influences the photocatalytic efficiency through structural features, and the interaction between the vat dyes leads to the enhancement of contact between the surface, and irradiation likewise decreases with the amount of semiconductor required [179]. Thus, there are some studies focused on the importance of semiconductor supported zeolite for vat dye photodegradation, including Co-ZSM-5, TiO_2_-HZSM-5, Fe-exchange zeolite, and CuO-X zeolite [180, 181].

TiO_2_ has been the most studied material for the photocatalysis of vat dyes [182–186]. ZnO has also been identified to be the main contender whose physicochemical properties are comparable to those of TiO_2_. However, ZnO, just as with TiO_2_, suffers from its large band gap energy that is close to 3.2 eV, which limits its adsorption of solar light emission that reaches the earth to less than 3 to 4%. For both semiconductor materials, the valence band is composed of O^2−^ (2p orbital), which is of anionic character, that induces interactions and oxidation composition with the vat dyes (alkali metal salts) [187–190]. However, this anionic character gains rapid recombination and holes during the oxidation process and in turn diminishes the efficiency of the photocatalytic reaction [191, 192]. From this viewpoint, efforts have been allocated to extend the adsorption of TiO_2_ and ZnO to deal with the photosensitization by vat dye molecules. According to the literature, reports on ZnO for the vat dye photodegradation are still scarce. The nanosized ZnO has extracted intense interest in recent years, especially in vat dye photodegradation for enhancing its performance, such as changes in surface properties and increase in surface area as well as in quantum effects of the overall decolorization process [193–195]. Upon light irradiation, this nanosized ZnO produced a highly active radical species that can quickly oxidize the vat dyes into harmful residues.

Copper sulfide (CuS) is another type of semiconductor that is currently used in photocatalytic decolorization of vat dyes [196–199]. CuS with a layered structure is a transparent p-type semiconductor with a band gap above 3.1 eV [198]. The top of the valence band is principally composed of well-hybrid state of Cu 3d and S-3p states, while the bottom of the conduction band consists primarily of Cu 4s state [196, 197]. The band gap of CuS was recognized to be a direct-allowed transition type through the analysis of the symmetry [199]. This small dispersion of the conduction band leads to the broadband gap and high stability of CuS to be more convenient for vat dye adsorption and oxidation.

### 2.5. Photocatalytic Decolorization of Azoic Dyes

The azoic dyes that are normally used on industrial scale have characteristics that are dependent on one or more azo bonds (–N=N–) with aromatic rings [167, 200–202]. The aromatic ring system of the dyes helps to strengthen the Van-der-Waals forces between dye and fibers [203]. Most synthetic dyes have significant structural variations and are extremely stable in performance under light and washing and most severely are resistant to aerobic biodecolorization by bacteria [204, 205]. Thus, it has been reported that the effluents from textile or dye industries involve aromatic compounds which are chemically stable and harmful to human health [201]. Various substitutions on the aromatic nucleus and most versatile groups of compounds give structurally diverse which make them recalcitrant, xenobiotic, noticeable in public, teratogenic, and resistant to degradation [204]. The amount of azo dye concentrations present in wastewater varied from very low to high concentrations (5 to 1500 mgL^−1^) that leads to color dye effluents causing toxicity, including carcinogenic and mutagenic effects in biological ecosystems. In addition, under anaerobic conditions acid dyes are promptly decreased to potentially hazardous aromatic amines [206]. Therefore, water soluble azo dyes even at low concentrations can cause water streams to be highly colored. On the other hand, azo dyes are insoluble in water but may become solubilised by alkali reduction, for instance, by sodium dithionite which is a reducing agent in the presence of sodium hydroxide [207]. Hence, they tend not to contain several functional groups which may be assailable to oxidation and reduction, which in turn gives harmful effects to the water stream habitat.

The biological approach of the decolorization of azo dyes takes place either by adsorption on the microbial biomass such as fungi, algae, yeast, and bacteria, along with anaerobic to aerobic treatments or biodegradation by the cell [208]. Azo dyes can also be reduced chemically by sulfide and dithionite. The decolorization mechanism of azo dyes based on the extracellular chemical reduction with sulfide was postulated for sulfate reducing environments [209]. However, it has also been noted that for the treatment of azo dyes containing wastewater, traditional methods like flocculation, adsorption onto activated carbon, activated sludge process, and reverse osmosis have difficulties in complete degradation of pollutants and also have the further disadvantage of resulting in secondary pollution [208–210]. Moreover, anaerobic decolorization of azo dyes may also produce carcinogenic aromatic amines.

**Figure 6:**
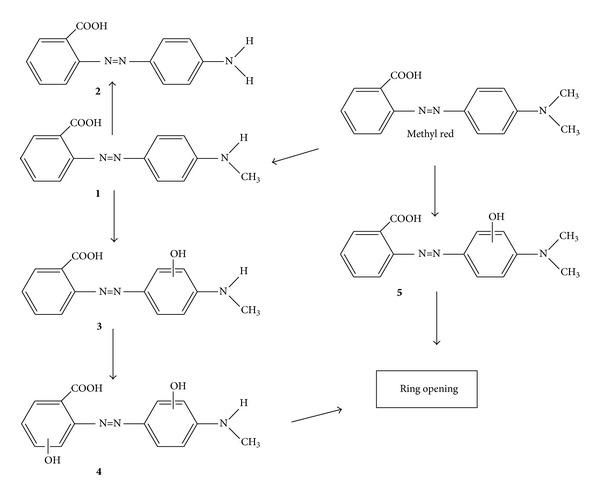
Proposed pathway for the photodecolorization of methyl red [216].

**Figure 7:**
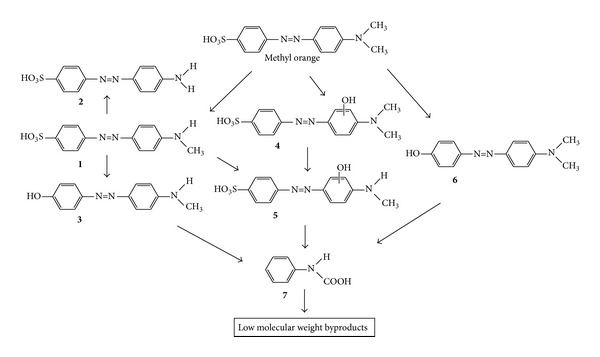
Proposed pathway for the photodecolorization degradation of methyl orange [216].

Therefore, the photocatalytic oxidation technique has received significant attention for destroying of azo dyes in recent years. This technique can be divided into homogenous and heterogeneous subgroups, based on the action of OH^•^ which enables almost complete mineralization of azo dyes under mild experimental conditions due to the high oxidation potential [211–215]. In heterogeneous photocatalysis of azo dyes, the electron-hole pairs will be initially produced by irradiation of a semiconductor with a photon of energy equivalent to or greater than its band gap width [213]. The electrons and holes may migrate to the semiconductors on the catalyst surface where they take part in redox reactions with the adsorbed azo dyes [212]. The oxidizing radicals could attack the azo dye molecule and disintegrate it into CO_2_ and H_2_O molecules which are nontoxic [212–214]. It has been suggested that the formation of free radicals acts as a primary oxidizing species [216]. The mechanism on photodecolorization of azo dyes with methyl red and methyl orange as a model of compound is illustrated in Figures 6 and 7, respectively.

It is claimed that azo dyes are noted for their photocatalytic decolorization in the absence of oxygen whenever a suitable electron donor or hydrogen source is present [217]. Structurally, azo dyes are double bonded belonging to different chromophoric groups and are heterocyclic and adsorb visible light [208]. The reduction of the chromophoric group shifts the visible region of the ultraviolet or infrared region, and thus a reduction in color is achieved. Consequently this phenomenon has encouraged several research works using heterogenous semiconductor photocatalysts like TiO_2_, ZrO_2_, SnO_2_, Fe_2_O_3_, CuO, ZnO, and CdSas as an alternative to conventional methods for the degradation of azo dyes from wastewater streams [218–220]. The degradation of hydrophobic and hydrophilic azo dyes has been demonstrated to be effective in acetone solution under exposure to UV light.

Recently, photocatalysis of azo dyes using solar or artificial light and TiO_2_ has been the objective of several studies as it is an attractive low energy strategy that has been applied to many other organic compounds (e.g., phenol) [4]. TiO_2_ is chemically inert, corrosion resistant, and most importantly, it works under mild conditions without any chemical additives [221, 222]. Meanwhile, it was found that in degradation of methyl orange or 4-4-[(dimethylamone)phenylazo] benzenesulfonic acid, a TiO_2_ film was up to 50% less effective than the TiO_2_ slurry. However, some improvements were observed after coating/doping the TiO_2_ film with metals, but the films were still not as impressive as the slurry [223, 224]. Meanwhile, other studies have shown that only cationic azo dyes can be adsorbed on the surface of the photocatalyst and simultaneously their photocatalytic degradation was quicker than the degradation of anionic azo dyes like Eriochrome Black T [225, 226]. It has been found that TiO_2_ adsorbed almost only cationic azo dyes, except for the anionic Quinizarin with an adsorption efficiency of 21.8% [227, 228]. Apart from photooxidation, the photoreduction of azo dyes is also known as a significant decolorizing or a folding pathway. This fact can be explained in relation to the surface structure of TiO_2_. In the unmodified surface structure of crystal TiO_2_, oxygen atoms are mainly present with a high electron density which creates a negative center [228–230]. Thus, the TiO_2_ particles have a negative charge and are more suitable to adsorb cationic azo dyes than the ones with anionic characteristics [182, 231, 232]. Furthermore, the modelling of photodecoloration of nonbiodegradable azo dyes was investigated recently with Reactive Red 2 in a cocktail mixture of triethylamine and acetone. It was found that the cocktail photolysis system was able to entirely decolorize the azo dye in a short time and the overall dye removal followed pseudo-first-order decay kinetics [233].

Furthermore, a TiO_2_-based photocatalysis for azo dye degradation has been developed. It can be applied as a film and has the effectiveness of the slurry [234–236]. An approach to enhance the photocatalytic reaction rate is by modifying the semiconductor with transition metal. Decorating TiO_2_ with other metal/nonmetal or metal/metal combinations can decrease its band gap and allow for activation by the longer wavelength of visible light [237–239]. Hence, solar energy can be used more effectively in the photocatalysis process. Currently, many metals (e.g., Fe, Cu, Co, Al, Cr, Ce, Ag, and Nd) and nonmetals (N, C, F, S, and B) have been attached onto TiO_2_ for azo dye degradation [237–242]. Among the metals, Ag^+^ has been recognized to be more effective than Fe^3+^, Co^2+^, Ce^4+^, and Cu^2+^, since it traps the photogenerated electrons and avoids the recombination of electrons and holes [243].

ZnO has been demonstrated to have a much higher efficiency than TiO_2_ in the case of azo dyes degradation irradiated by UV light; however, studies on heterojunction systems applied to water treatment have primarily been restricted to the sensitization of TiO_2_ [244, 245]. This statement has been supported by the fact that ZnO has numerous advantages over TiO_2_. This includes high efficient photocatalytic activity, and photodegradation of diluted azo dyes cannot proceed sufficiently because of insufficient contact between azo dyes and semiconductors. This is an important factor in hindering photocatalytic activity [246]. The mass transfer from azo dyes to the semiconductor surface limits the photodegradation rate of diluted azo dyes. It is important that visible light degradation of some dyes utilizing ZnO was shown to be more effective than TiO_2_. In this case the degradation mechanism was based on electron injection from the exited dyes to the ZnO conduction band. This was much more significant as compared to TiO_2_ which indicates high efficiency of charge transport and limited charge loss [247–249].

### 2.6. Photocatalytic Decolorization of Sulfur Dyes

Textile industries generate large amounts of colored sulfur dye effluents which are toxic and induce a lot of damage to the environment. In view of the mutagenic character or carcinogenic nature of sulfur dyes, the deleterious effects of the color in receiving water, and the customary resistance of the sulfur dyes to biological degradation, the necessity of investigating new alternatives for appropriate treatment of this kind of dyes is evident [250–252]. Thus, various methods for the removal of sulfur dyes have been reported, including biological and chemical flocculation, coagulation, adsorption and oxidation, electrochemical oxidation, membrane separation, and ion exchange methods [253–255]. These methods have their own limitations for the removal of sulfur dyes, including being expensive, time consuming, and commercially unattractive as well as resulting in the production of secondary wastes [254]. Furthermore, these processes are also ineffective for sulfur dye removal since sulfur dyestuff is biorecalcitrant. In addition, these series of physicochemical treatments prepare only a phase transfer of sulfur dyes and produce huge quantities of sludge [256, 257].

The efficiency of a photocatalytic decolorization reaction is determined by the properties and quality of the photocatalyst, which is often a semiconductor with the ability to create electron-hole pairs under photoillumination [258, 259]. Thus, it is an important step to recognize an efficient and suitable photocatalyst during the decomposition process. In recent decades, different mixed metal oxides consisting of TiO_6_, TaO_6_, or NbO_6_ octahedral units, such as BaTi_4_O_9_, SrTiO_3_, K_4_Nb_6_O_17_, InTaO_4_, and Ni_*x*_TaO_4_ had been extensively investigated as a new class of photocatalysts in the field of sulfur dye degradation [260–262]. These kinds of photocatalysts belong to a family of uniform heterogenous catalysts [261]. Yet only a few of these photocatalysts have been studied for the removal of environmental contaminants, and earlier authors have all used the solid-solid blending method to synthesize their sample. Recently, the typical photocatalysts developed are mostly oxides containing d-block element ions as Ti^4+^, Ta^5+^, Nb^5+^, and Zr^4+^ with d0 electron configuration [263]. Very recently, researches have also focused on p-block metal oxide photocatalyst with d10 electron configuration due to their fair mobility for sulfur dye degradation [261, 262]. TiO_2_ was found to be the most efficient photocatalyst for photodegradation of sulfur dyes because of faster electron transfer of molecular oxygen [264–266]. Furthermore, TiO_2_ photocatalyst is largely available as a nontoxic, inexpensive, and with relatively high chemical stability [42]. It has been noted that the photocatalytic degradation of sulfur dyes in solution is initiated by photoexcitation of the semiconductor, followed by formation of an electron-hole pair on the semiconductor surface [267]. The high oxidation potential of the hole in the semiconductor permits the direct oxidation of sulfur dyes into reactive intermediates [268, 269]. Highly reactive OH^•^ can also be formed either by decomposition of water or by the reaction of the hole with OH^−^. The OH^•^ radical is a very strong, nonselective oxidant that leads to the degradation of organic chemicals [270].

There are certain relationships between properties of dyes and treatment mechanisms. Sulfur dyes are often made of azo compounds, sulfide structures, or anthraquinones, and they have several –C=O, –NH–, and aromatic groups. These dyes tend to be adsorbed by Fe(OH)_*x*_ particles [271–273]. However, the photodegradation of sulfur dyes utilizing semiconductors is not new. The sulfur dye treatment of photocatalyst would be more suitable if the semiconductor was immobilized, so the semiconductors would not have to be separated from the sulfur dye solution [274, 275]. Thin films are one of the most important technological applications. Thin film photocatalyst towards sulfur dyes photodegradation offers high stability and convenient reuse and hence has received more and more attention [276]. Furthermore, photocatalysis supports such as zeolite have been extensively used to enhance the photodegradation of sulfur dyes. Zeolites are crystalline aluminosilicates with cavities in which the size can change in the range from one to several tens of nanometers depending on the type of aluminosilicate framework, Al/Si ratio, and the origin of the ion exchange cations [277–279]. These characteristics of zeolite make it more selective for photocatalytic oxidation and are crucial especially when using environmentally benign oxidants.

## 3. Recent Advances in Synthetic Dyes Photocatalytic Decolorization

Industrial effluent detoxification is one of the most challenging global problems. Dyes, phenols, pesticides, fertilizers, detergents, herbicides, surfactants, and other synthetic organic compounds are disposed of directly into the environment, without being treated, controlled, or uncontrolled, without an effective treatment strategy [280–282]. Their toxicity, stability to natural decomposition, and persistence in the environment have been the cause of much concern to societies and regulatory authorities around the world [283, 284].

Although the strong potential of photocatalytic process for wastewater treatment is widely recognized via numerous patents and publications, technical development at industrial level has not been met with much success [285–287]. This is due to the high operating cost of the photocatalytic oxidation process relative to existing biological treatments [288]. Since in tropical countries, sunshine is available in abundance; therefore, application of this oxidation technology using solar light can be a cost- and energy-effective detoxification technology. Furthermore, the limitations of the photocatalyst system can be addressed in terms of the tight range of pH in which the reaction proceeds, the requirement for recovering the precipitated catalyst after treatment, and the deactivation by some ion-complexing agents such as phosphate anions [289, 290].

Using solar energy is an interesting aspect in photocatalyst technologies. Solar photocatalysis has become an important area of research in which sunlight is the source of illumination to perform various photocatalytic reactions with regard to different kinds of dyes [291, 292]. As visible light is the main component of solar radiation, the development of a stable photocatalytic system, which can be affected by visible light, is most probably indispensable. In order to overcome the limitations, many studies on coupled semiconductor photocatalysts like ZnO-TiO_2_, CuO-ZnO, CuO-TiO_2_, CuO-SnO_2_, TiO_2_-SnO_2_, ZnO-SnO_2,_ and so on have been reported [198, 293–295]. These series of binary oxide photocatalysts showed enhanced catalytic activities and selectivities compared to the monocomponent photocatalyst. This combined system also provides a more controllable rate of recombination as the composition of two semiconductors with different band gaps can suppress the recombination of e^−^/h^+^ pairs [296]. Amongst the series of binary systems, CdS/TiO_2_ showed the most prospect as an effective visible light photocatalyst for dye reduction and degradation. In the system of TiO_2_/CdS, the photogenerated electrons in CdS are transferred into the TiO_2_ particle, while the holes remain in the CdS particles [297, 298]. This combination has also overcome the limitation of native CdS as photocatalyst due to its photocorrosion. Other researchers have loaded semiconductor with carbon-based nanomaterials like activated carbon, CNTs, graphene, graphite, and other matrices to improve the photocatalytic activity or cycling and its ratings performance [299–302]. Meanwhile, recent research has indicated that organic polymer films such as chitosan and cellulose films can ensure the stabilization of semiconductors especially in nanosized form and also provide an interface for the charge transfer and correspondingly improve photocatalytic efficiency [301–303]. In addition, the incorporation of such biopolymers assist in reducing the leakage of semiconductor particles in treated water during the dye removal and degradation, since those types of biopolymers are effective adsorbents and chelators for semiconductor ions in aqueous solutions [304].

## 4. Influence of Dye Type on the Photocatalytic Process

The chemical structure of the organic dyes has a considerable effect on the reactivity of dyes on photodegradation system [301]. This effect has been explored by different researchers. For example, the COD removal rate of RY17 was found to be higher than RR2 and RB4 dyes. This is due to the structural difference among the three molecules of dyes. RY17 and RR2 are equipped with an azo group (–N=N–), which is not present in RB4 molecules and suspected to photodegradation. In addition, –CH_2_–OS_2_– linkage in RY17 is also labile in the reaction environment. In RB4, the presence of anthraquinone structure and the absence of azo band make it resistant to photodegradation [305].

Meanwhile, the removal of reactive orange 16 was maximum, closely followed by reactive blue 4 and reactive 5 in case of TiO_2_ photocatalysis. It may be due to the difference in chemical structure of dyes, resulting in difference in adsorption characteristics and difference in susceptibility to photodegradation [43, 44, 47]. The chemical structure of the dyes indicates that reactive black 5 has more complex structure, making it less photodegradable. Another reason may be due to absorption of light photon by dye itself leading to a less availability of photons for hydroxyl radical generation. It was observed from the absorption spectra of three dyes in near UV range that reactive black 5 strongly absorbs near UV radiation compared to reactive orange 16 and reactive blue 4, leading to less by the dye molecules is thought to have an inhibitory effect on the photogeneration of holes or hydroxyl radicals, because of the lack of any direct contact between the photons and immobilized TiO_2_. [48]. Indeed, it causes the dye molecules to adsorb light and the photons never reach the photocatalyst surface; thus, the photodegradation efficiency decreases.

It is also important to notice that degradation pathway of organic dyes may be different as according to the chemical structure and functional groups. For example, with an addition of a ^•^OH radical to an aromatic ring of dyes molecules, a labile H atom is produced [56–60]. This mechanism is also unsatisfactory for hydroxy azo dyes (AO7 and AO8). In that case, abstraction of the H atom, carried by an oxygen atom in the azo form and by a nitrogen atom in the hydrazone form, competes with the addition of ^•^OH radical on a phenyl or naphthyl nucleus [72].

The functional groups in the chemical structure of dye could be nitrite groups, alkyl side chain, chloro group, carboxylic group, sulfonic substituent, and also hydroxyl groups [305]. The appropriate photocatalyst material has to be chosen depending on these functional groups in the chemical structure of dye [81–84]. Every group that tends to decrease the solubility of molecules in water will decrease the degradation process. In order to evaluate the influence of a nitrite group, the degradation of an analogous pair of dyes such as Acid Red 29 and Chromotrope 2B can be mentioned. Chromotrope 2B contains a nitrite group in the para position with respect to the azo function [305]. This substituent interacts with the phenyl ring and there is a consequent delocalization of the p electrons of the ring and of the unpaired electrons of the heteroatom. As a result, the phenyl ring is electron-enriched, and the nitrite group thus favors attack of an electrophilic entity. The experiment confirms this hypothesis: Chromotrope 2B reaction rate is slightly higher than that of AR29. Hydroxyl radicals have a very short lifetime, so that they can only react where they are formed [72]. Therefore, oxidation reactions can only be successfully performed in homogeneous media. As it was previously mentioned, every group that tends to decrease the solubility of molecules in water will decrease the degradation process. This explains why the rate of decomposition clearly decreases with increasing length of the side chain and consequently with increasing hydrophobicity of the dye molecule, as seen at the degradation of AB25 and RB19 [81–85]. A parallel reaction may take place between ^•^OH radical and hydrogen atoms of the side chains. This reaction competes with destruction of the dye chromophore, without leading to a decrease in the absorbance of the solution.

Considerable decrease of photocatalytic decolorization rate was observed when two or three chloro substituents were present on the phenyl ring of a pyrazolone dye [104, 170]. Indeed, comparison of acid yellow 17 and acid yellow 23 decolorization rates suggests that the difficulty of the dye to be degraded directly depends on the number of electron withdrawing chloro groups in the molecule. The decolorization kinetics of acid yellow 17 is less than those of acid yellow 23.

The photocatalytic decolorization of four organic dyessuch as Alizarin S, Orange G, Methyl Red, and Congo Red by UV/TiO_2_ has been processed to explore the effect of the presence of carboxylic substituent in dye chemical structure. The photocatalytic rate constants were in the following order: Methyl Red > Orange G *≈* Alizarin S > Congo Red [274]. It has been explained that the higher degradability of MR could be due to the presence of a carboxylic group which can easily react with H^+^ via a photo-Kolbe reaction. However, the presence of a withdrawing group such as –SO_3_^−^ is probably at the origin of the less efficient Orange G and Alizarin S degradations [274]. Another suggestion to explain the different reactivity of these dyes could also be their ability to adsorb on TiO_2_ surface.

Unexpectedly, the presence of the more powerful electron withdrawing sulfonic group on a molecule makes it only very slightly less sensitive to oxidation. Indeed, molecules with one, two, or three sulfonic functions have almost the same reactivity with respect to oxidation by hydroxyl radicals [63, 83, 169, 209]. Acid red 14 containing two sulfonic groups is more reactive in a photocatalytic degradation process in comparison with acid red 18 and acid red 27 that contain three sulfonic substituents [169]. Study of the influence of the sulfonic group is very difficult, because this substituent operates in different fields: it decreases electron density in the aromatic rings and the *β* nitrogen atom of the azo bond by −I and +M effects. On the other hand, it increases the hydrophilic-lipophilic balance of the dye molecules and consequently slows down their aggregation degree [63, 209].

The electronic properties of a hydroxyl group are −I and +M effects. That is why the photocatalytic decolorization rate of acid red 29, which contains two hydroxyl substituents, is more than that of orange G, which contains one hydroxyl substituent [83]. In both dyes, one molecule contains a hydroxyl group next to the azo bond. But the resonance effect of a substituent operates only when the group is directly connected to the unsaturated system. Therefore, to explain the effect of the hydroxyl group on the reactivity of the organic matter, only the field effect (−I) must be considered. The number of hydroxyl groups in the dye molecule can intensify this resonance and, consequently, the degradation rate of the dye [209].

Photocatalytic decolorization rate of monoazo dyes is higher than dyes with anthraquinone structure. The presence of methyl and chloro groups in the dye molecule decreases slightly the process efficiency while a nitrite group acts in an opposite direction [305]. Alkyl side chain decreases the solubility of molecule in water and consequently disfavors the photocatalytic degradation process. The dyes which contain more sulfonic substituents are less reactive in the photocatalytic process, while hydroxyl group intensifies the electron resonance in the molecule and the degradation rate of the dye. Photocatalytic decolorization takes place at the surface of the catalyst. Dye molecules adsorb onto the surface of photocatalyst material by electrostatic attraction and get mineralized by nonselective hydroxyl radicals. Therefore, the adsorption of the target molecule on photocatalyst material surface may be regarded as a critical step toward efficient photocatalysis.

## 5. Conclusion

In the textile industry, regulations concerning the discharge of wastewater have become more and more stringent. The synthetic dyes utilized in the textile and other industries generate hazardous waste. The dye is utilized to impart color to materials of which it becomes an integral part. However, dye removal is an important but challenging area of wastewater treatment since some dyes and their degradation products are carcinogenic and toxic to mammals. Destructive oxidation of poisonous dyes via photocatalytic approaches have recently received considerable attention since colored aromatic compounds have proven to be degraded effectively by a variety of heterogenous semiconductor catalysts. Photocatalysis aims at mineralization of poisonous dyes to CO_2_, H_2_O and inorganic compounds or at least their transformation into biodegradable or harmless products. Finally, taking into account that UV light is not only expensive but also harmful to aquatic life, there is the need to improve the ability of photocatalysts to work with visible light.

## Figures and Tables

**Table 1:** Usage and characterization of dyes.

Group of dyes	Characteristics	Application	References
Direct dyes	(i) Dyeing process with one action, without the assistance of an affixing agent; simplest and cheapest dyes (ii) Water soluble anionic dyes; substantive to form aqueous media in the electrolytes (iii) High affinity for cellulose fibers (iv) Apply to the dye materials to improve wash fastness properties (chelation with salts of metals and treatment with a cationic dye-complexing resin or formaldehyde) (v) Some contain sulphonate functionality to improve solubility (negative charge of dyes and fibers repel each other) (vi) Its flat length enable and shape to lie along-side cellulose fiber and maximum (vii) Van-der-Waals, hydrogen bonds, and dipole (ix) Dyeing method: exhaust/beck/continuous	Cotton, cellulosic, regenerated cellulose, paper, leather, nylon, and blends	[6, 7]

Vat dyes	(i) Water insoluble dyes (ii) Apply as soluble leuco salt after reduction in an alkaline solution with sodium hydrogen sulfide (iii) The leuco form is reoxidized to the insoluble keto form to redevelop the crystal structure (iv) More chemically complex (v) Dyeing methods: exhaust, package, continuous	Cotton, linen and rayon, soap	[8, 9]

Organic pigments	(i) Negatively charged compounds (ii) Made from ground up colored rocks, minerals, animals, and plants (iii) No chemical information (iv) Classification based on the dye's source and color (v) Application requires a mordant	Cotton, paper, cellulosic, blended fabrics	[10, 11]

Reactive dyes	(i) React directly with the fiber molecules to form chemical bonds (ii) Conceivable to achieve very high wash fastness properties (iii) Require facile dyeing methods (iv) Simple chemical structure (v) The largest dye class (vi) Adsorption spectra with a narrow adsorption band (vii) Dyeing is bright (viii) Dyeing methods: exhaust, beck cold pad batch, and continuous	Cellulosic fabric and fibers	[12, 13]

Dispersed dyes	(i) Water insoluble nonionic (ii) Require additional factors (dye carrier, pressure, and heat) to penetrate synthetic dyes (iii) Dispersed in aqueous media wherever the dye is dissolved into fibers (iv) Especially on polyester and to a lesser extent on cellulose acetate, nylon, acrylic fibers, and cellulose (v) Niche market in dye diffusion thermal transfer process for electronic photography and thermal transfer printing (vi) Dyeing method: high temperature exhaust, continuous	Synthetic/hydrophobic fibers from aqueous dispersion	[14, 15]

Acid dyes	(i) Water soluble anionic dyes (ii) Typical pollutants: color, organic acid, unfixed dyes (iii) Dyeing methods: exhaust, beck, and continuous	Silk, wool, synthetic fibers, leather, nylon, modified acrylics, paper, ink-jet printing, food, cosmetics	[16, 17]

Azoic dyes	(i) Contain one azo group (mono azo), two azo group (disazo), three azo (trisazo), four azo group (tetrakisazo), or more (polyazo) groups (ii) Attach to two classes of which at least one but usually both are aromatic (iii) Exist in the transform 1 in (iv) which the bond angle is 120° and the nitrogen atoms are sp^2^ hybridized (v) Consist of electron accepting substituents and electron donating substituents (vi) Named as carbocyclic azo dyes if include only aromatic groups (naphthalene and benzene) (vii) Named as heterocyclic azo dyes if include heterocyclic group	Printing inks, pigments	[18, 19]

Basic dyes	(i) Water soluble cationic dyes (ii) Can be applied directly to cellulosic with no mordants (or metal-like copper and chromium) (iii) Yield colored cations in solutions (iv) Apply as brightness of shade is more important than fastness to washing and light (v) Some basic dyes show biological activity and are used in medicine as antiseptics (vi) Salt-forming counter ion (vii) Colorless anion of a low molecular mass, organic, or inorganic acid (viii) Can be turned to water soluble dye bases by addition of alkali (ix) The positive charge is localized on an ammonium group (x) Dyeing methods: exhaust, beck, and continuous	Silk, wool, cotton, polyacrylonitrile, modified nylons, modified polyester, tannin-mordanted cotton	[20, 21]

Oxidation dyes	(i) Primarily aromatic compounds that belong to three major chemical families (Diamines, Aminophenols (amino naphthols) and Phenols or naphthols) (ii) Colorless and are typically a low molecular weight product (iii) Categories-oxidation base as a primary, intermediate and coupler as a secondary, intermediate	Hair	[22, 23]

Developed dyes	Any group of direct azo dyes which after applying to the fiber can be diazotized further and coupled on the fiber to form shades faster to washing	Cellulosic fibers, fabric	[24, 25]

Mordant dyes	A substance utilized to set dyes on fabrics or tissue sections by forming a coordination complex with the dye that attaches to the tissue or fabric	Cellulosic fibers, fabric, silk, wool	[26–28]

Optical/ fluorescent brightener	(i) Absorb light in the violet region and ultraviolet (mostly 340–370 nm) of the electromagnetic spectrum, and reemit light in the blue region (usually 420–470 nm) (ii) Utilized to increase the appearance of color of paper and fabric, causing a “whitening” effect, making materials look less yellow by increasing the overall amount of blue light reflected	Synthetic fibers, leather, cotton, sport goods	[29–31]

Solvent dyes	(i) Water insoluble (ii) Free of polar solubilizing groups such as carboxylic acid, sulfonic acid, or quaternary ammonium	Wood staining, solvent inks, waxes, coloring oils, plastic, gasoline oil	[32, 33]

Anthraquinone	(i) The oldest dyes (4000 years) (ii) No natural counterpart (iii) Low cost effectiveness	Wrapping of mummies	[34]

Indigoid	(i) Expensive (ii) Made of tyrian purple (iii) Give progressively paler blue shades (iv) Oxidation process of indigoid gives phenylacetic acid	Textile, wool, linen, cotton Use exclusively for dyeing denim jeans, jackets	[35]

Sulfur dyes	(i) Made by heating aromatic or heterocyclic compounds with species that release sulfur or sulfur (ii) Classified by sulfur bake, polysulfide melt dyes, and polysulfide bake (iii) Not well-defined chemical compounds (iv) Mostly contain various thiophenolic and heterocyclic sulfurs (v) On oxidation, the monomeric molecules cross-linked into large molecules form disulfide bridge (vi) Dyeing methods: continuous	Cotton, other cellulosic	[36]

**Table 2:** Types of adsorbents used with different anionic/acid dyes.

Adsorbent	Anionic dyes	References
Organo-bentonite	Acid scarlet	[72]
	Acid turquoise blue	[73]
	Indigo carmine	[74]

Ammonium functionalized mesoporous materials	Reactive brilliant red	[75]
	Acid fuchsine	[76]
	Orange IV	[77]
	Methyl orange (MO)	[78]

Apatitic tricalcium phosphate	Reactive yellow 4	[79]
Apatitic octocalcium phosphate		[80]

Wood shaving bottom ash	Red reactive 141	[81]

Bagasse ash	Acid blue 80	[82]
